# Mutations in the β-subunit of rod phosphodiesterase identified in consanguineous Pakistani families with autosomal recessive retinitis pigmentosa

**Published:** 2011-05-25

**Authors:** Shahbaz Ali, S. Amer Riazuddin, Amber Shahzadi, Idrees A. Nasir, Shaheen N. Khan, Tayyab Husnain, Javed Akram, Paul A. Sieving, J. Fielding Hejtmancik, Sheikh Riazuddin

**Affiliations:** 1 National Centre of Excellence in Molecular Biology, University of the Punjab, Lahore, Pakistan; 2 The Wilmer Eye Institute, Johns Hopkins University School of Medicine, Baltimore, MD; 3 Allama Iqbal Medical College, University of Health Sciences, Lahore, Pakistan; 4 Ophthalmic Genetics and Visual Function Branch, National Eye Institute, National Institutes of Health, Bethesda, MD

## Abstract

**Purpose:**

This study was designed to identify pathogenic mutations causing autosomal recessive retinitis pigmentosa (RP) in consanguineous Pakistani families.

**Methods:**

Two consanguineous families affected with autosomal recessive RP were identified from the Punjab Province of Pakistan. All affected individuals underwent a thorough ophthalmologic examination. Blood samples were collected, and genomic DNAs were extracted. Exclusion analysis was completed, and two-point LOD scores were calculated. Bidirectional sequencing of the β subunit of phosphodiesterase 6 (*PDE6β*) was completed.

**Results:**

During exclusion analyses both families localized to chromosome 4p, harboring *PDE6β*, a gene previously associated with autosomal recessive RP. Sequencing of *PDE6β* identified missense mutations: c.1655G>A (p.R552Q) and c.1160C>T (p.P387L) in families PKRP161 and PKRP183, respectively. Bioinformatic analyses suggested that both mutations are deleterious for the native three-dimensional structure of the PDE6β protein.

**Conclusions:**

These results strongly suggest that mutations in *PDE6β* are responsible for the disease phenotype in the consanguineous Pakistani families.

## Introduction

Retinitis pigmentosa (RP) is one of the most common retinal hereditary disorders, affecting approximately 1 in 5,000 people worldwide [1]. It is a progressive rod–cone dystrophy that manifests with nyctalopia, gradual degeneration of visual fields, and eventual decline of visual acuity. Clinical symptoms include pigmentary deposits in the retina, waxy disc pallor, and attenuation of retinal blood vessels [2]. RP is genetically a heterogeneous disease that is inherited as an autosomal dominant, autosomal recessive, as well as an X-linked trait [3]. To date, more than 50 loci and genes have been implicated in the pathogenicity of autosomal recessive RP.

Phosphodiesterase 6 (PDE6) is an important component of the visual cascade. It consists of two catalytic subunits, namely, *PDE6α* and *PDE6β,* and a regulatory subunit, *PDE6γ* [4]. PDE6 mediates the hydrolysis of cyclic guanine monophosphate (cGMP), and regulation of cGMP levels is essential for the phototransduction cascade as prolonged imbalance in cGMP metabolism can potentially disrupt the visual signaling pathway [5]. *PDE6β* encodes the β subunit of PDE6 and has been implicated in the pathogenesis of autosomal dominant RP [6], autosomal recessive RP [7], and congenital stationary night blindness [8]. According to the human gene mutation database, 21 mutations in *PDE6β* have been identified in patients with autosomal recessive RP (arRP).

Here, we report two consanguineous families from the Punjab province of Pakistan that were diagnosed with arRP. Clinical records available to us are suggestive of an early, most probably a congenital, onset. Exclusion analysis revealed linkage with significant logarithm of odds (LOD) scores to chromosome 4pter-p16.2, a region that harbors *PDE6β*. Sequencing of *PDE6β* identified missense mutations that were not present in ethnically matched control chromosomes.

## Methods

### Clinical assessment

One hundred and twenty-five consanguineous Pakistani families with nonsyndromic arRP were recruited to participate in a collaborative study between the National Center of Excellence in Molecular Biology, Lahore, Pakistan, and the National Eye Institute, Bethesda, Maryland, to identify new disease loci causing inherited visual diseases. Approval of the Institutional review board was obtained for this study from both institutes, and the participating subjects gave informed consent consistent with the tenets of the Declaration of Helsinki. A detailed pedigree drawing and medical history was obtained by interviewing family members. Funduscopy was performed at Layton Rehmatulla Benevolent Trust Hospital, Lahore. Electroretinography (ERG) measurements were recorded with equipment manufactured by LKC (Gaithersburg, MD). Rod responses were determined through an incident flash attenuated by −25dB and the rod-cone response was measured at 0dB. Isolated cone responses were recorded at 0dB with a 30Hz flicker over a background illumination of 17–34 cd/m^2^. Approximately 10 ml of blood samples were drawn from affected and unaffected members of the family and stored in 50 ml Sterilin® falcon tubes (BD Biosciences, San Jose, CA) containing 400 μl of 0.5 M EDTA. Blood samples were kept at −20 °C for long- term storage.

### DNA extraction

DNA was extracted by a nonorganic method as described previously [9,10]. Briefly, aliquots of 10 ml blood samples were mixed with 35 ml of TE buffer (10 mM Tris-HCl, 2 mM EDTA, pH 8.0), and the TE–blood mixture was centrifuged at 1,800× g for 20 min. The supernatant was discarded, and the pellet was resuspended in 35 ml of TE buffer and centrifuged at 1,800× g for 20 min. The TE washing was repeated two to three times, and the washed pellet was resuspended in 2 ml of TE. A protein digestion cocktail of 6.25 ml (50 μl [10 mg/ml] of proteinase K, 6 ml TNE buffer [10 mM Tris HCl, 2 mM EDTA, 400 mM NaCl], and 200 μl of 10% sodium dodecyl sulfate) was added to the resuspended pellets and incubated overnight in a shaker (250 rpm) at 37 °C. The digested proteins were precipitated by adding 1 ml of 5 M NaCl, followed by vigorous shaking and chilling on ice for 15 min. The precipitated proteins were pelleted by centrifugation at 1,800× g for 20 min and removed. The supernatant was mixed with an equal volume of phenol/chloroform/isoamyl alcohol (25:24:1), and the aqueous layer containing the genomic DNA was carefully collected. The DNA was precipitated with isopropanol and pelleted by centrifugation at 2,400× g for 15 min. The DNA pellets were washed with 70% ethanol and dissolved in TE buffer. The DNA concentration was determined with a SmartSpec plus Bio-Rad spectrophotometer (Bio-Rad, Hercules, CA).

### Exclusion analyses

Exclusion analyses were performed with highly polymorphic fluorescent short tandem repeat (STR) markers spanning all known/reported loci for arRP. PCR for exclusion analyses were performed in a PCR System 9700 (Applied Biosystems). Briefly, each reaction was performed in a 5-μl mixture containing 40 ng genomic DNA, various combinations of 10 μM-dye-labeled primer pairs, 1× GeneAmp PCR buffer, 1 mM dNTP mix, 2.5 mM MgCl_2_, and 0.2 U of *Taq* DNA polymerase. Initial denaturation was performed for 5 min at 95 °C, followed by 10 cycles of 15 s at 94 °C, 15 s at 55 °C, and 30 s at 72 °C and then 20 cycles of 15 s at 89 °C, 15 s at 55 °C, and 30 s at 72 °C. The final extension was performed for 10 min at 72 °C and followed by a final hold at 4 °C. PCR products from each DNA sample were pooled and mixed with HD-400 size standards (Applied Biosystems). The resulting PCR products were separated in an ABI3100 DNA analyzer, and alleles were assigned using Genotyper Software version 3.7 (Applied Biosystems).

### Linkage analysis

Two-point linkage analyses were performed using the FASTLINK version of MLINK from the LINKAGE Program Package, whereas the maximum two-point LOD scores were calculated with ILINK from the LINKAGE Program Package [11,12]. arRP was analyzed as a fully penetrant trait with an affected allele frequency of 0.001. The marker order and distances between the markers were obtained from the Marshfield database and the NCBI chromosome 4 sequence maps. For the initial genome scan, equal allele frequencies were assumed, while for fine mapping, allele frequencies were estimated from 96 unrelated and unaffected individuals from the Punjab province of Pakistan.

### Mutation Screening

Primer pairs of individual exons of PDE6β were designed using the primer3 software. The sequences and annealing temperatures are shown in Table 1. Amplifications were performed in a 25-μl reaction containing 50 ng of genomic DNA, 8 pM of each primer, 250 μM dNTP mix, 2.5 mM MgCl_2_, and 0.2 U Taq DNA polymerase in the standard 1× PCR buffer provided by the manufacturer (Applied Biosystems). PCR amplification consisted of a denaturation step at 96 °C for 5 min, followed by 40 cycles, each consisting of 96 °C for 45 s followed by 57 °C (or specific annealing temperature of the primer pair) for 45 s and at 72 °C for 1 min. PCR products were analyzed on a 2% agarose gel, precipitated, and purified by ethanol precipitation. The PCR primers for each exon were used for bidirectional sequencing using Big Dye Terminator Ready reaction mix according to manufacturer instructions (Applied Biosystems). Sequencing products were resuspended in 10 μl formamide (Applied Biosystems) and denatured at 95 °C for 5 min. Sequencing was performed on an ABI PRISM 3100 Automated sequencer (Applied Biosystems). Sequencing results were assembled using ABI PRISM sequencing analysis software version 3.7 and analyzed using SeqScape software (Applied Biosystems).

**Table 1:** Primer sequences for PDE6β  exons.

**Exon**	**Forward primer**	**Reverse primer**	**Annealing temperature (°C)**
1a	CTGGTTTTCCTGGAAGGT	CTGGCGGTACATGAAGAG	55
1a	AGGATATGCAGGAGAGCAT	CTCCTCAGCACAGAACTAGC	55
2	TCTGCTGGACTGAGCACT	GCAGGTAAAGAGGTGGATG	55
3	GTGCACCTGAGCTTGTGTGT	ACCTACCCAGGTGAGCACAA	60
4	CCACAAGCTCAGATGAAACCT	ATCAGCACAGACCACACGTC	59
5	AAGGAGAAGGTGAGGCTTCC	CTGGTGGAGACCACAGACAG	59
6	GGAACACAGACTGGGAAGAC	AGTGAGTCGGCTTCTGTCTC	57
7–8	ACACACACGTGCAGCCTA	AGTGGCAAAAACGAATTCAC	57
9	AAACTCCAAATGCAGAGAGG	TGCTTCTGTGTGGGGTCT	57
10	AGACCCCACACAGAAGCACT	CTGTGACCCCTCAATGGAC	59
11	ACGGTCATTTGTCTCCAGAT	AGTCAGGCCCACTAAACATC	57
12	AACTGGGCAAGTTCTTCACT	TACTTCCCGTGTGCATTTTA	56
13	GAAGTCCAGGAGACGGTGT	AGGGGTTGGGATGACCTA	57
14	TACCAAGGGCAGCACTCA	CGCCACCATACACAGCTT	58
15	CAGGAGGTCAAGGCTGTATT	CACTGAGTGTCCAGGTCCTT	56
16	CCAAGGACCTGGACACTCA	GTGGGAGCAAGTGTGGAGA	59
17	CCTGGCCCTGTACTTCAA	CAAGGGCTACAGACCAATG	57
18	GAGGCTGAGGCACAAGAATC	ACTGCAGTACCCCCATCCTT	55
19	GGCAACGGACCATTGTTT	TGAGATAAGGACCCCACGAC	59
20	TCCATGAGCACATCTGAGTGA	TCCGGAAACTGATGTTCCTC	60
21	CGAGGTTTCTCCCTTCACAG	TGGCTCTGCTTTTCTCCATT	59
22	TGAGCATAATCAGGGCACAG	TTGGGCTTCCTAACCTCTTG	59

The amplification conditions are described in the Methods.

### Prediction Analysis

Evolutionary conservation of the mutated amino acid residues in other PDE6β orthologs was examined using the UCSC genome browser. The degree of evolutionary conservation of these amino acids and the possible impact of an amino acid substitution on the structure of PDE6β was investigated with PolyPhen and Sorting Intolerant From Tolerant (SIFT) tools available online [13,14].

## Results

**Figure 1:**
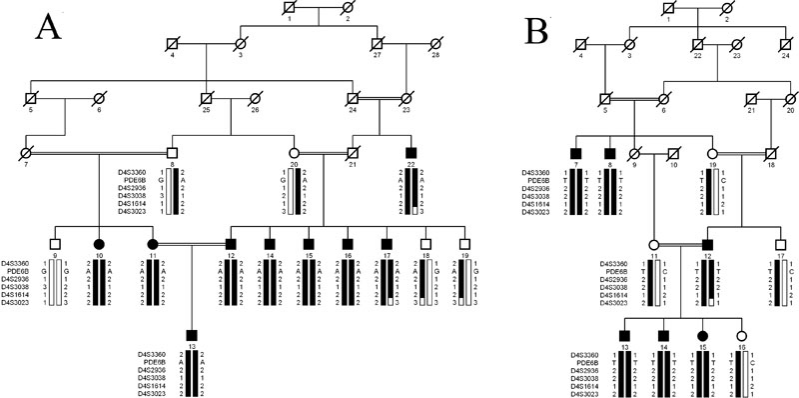
Pedigree drawings of families PKRP161 and PKRP183 with haplotyes of five adjacent chromosomes 4p microsatellite markers and variations identified in PDE6β. Single base changes: c.1655G>A and c.1160C>T were identified in PKRP161 and PKRP183, respectively and segregate with the disease phenotype in their respective families. Squares: males; circles: females; filled symbols: affected individuals; double lines between individuals: consanguineous mating; and a diagonal line through a symbol: a deceased family member. Alleles forming the risk haplotype are shaded black and alleles not co-segregating with retinitis pigmentosa are shown in white.

**Figure 2:**
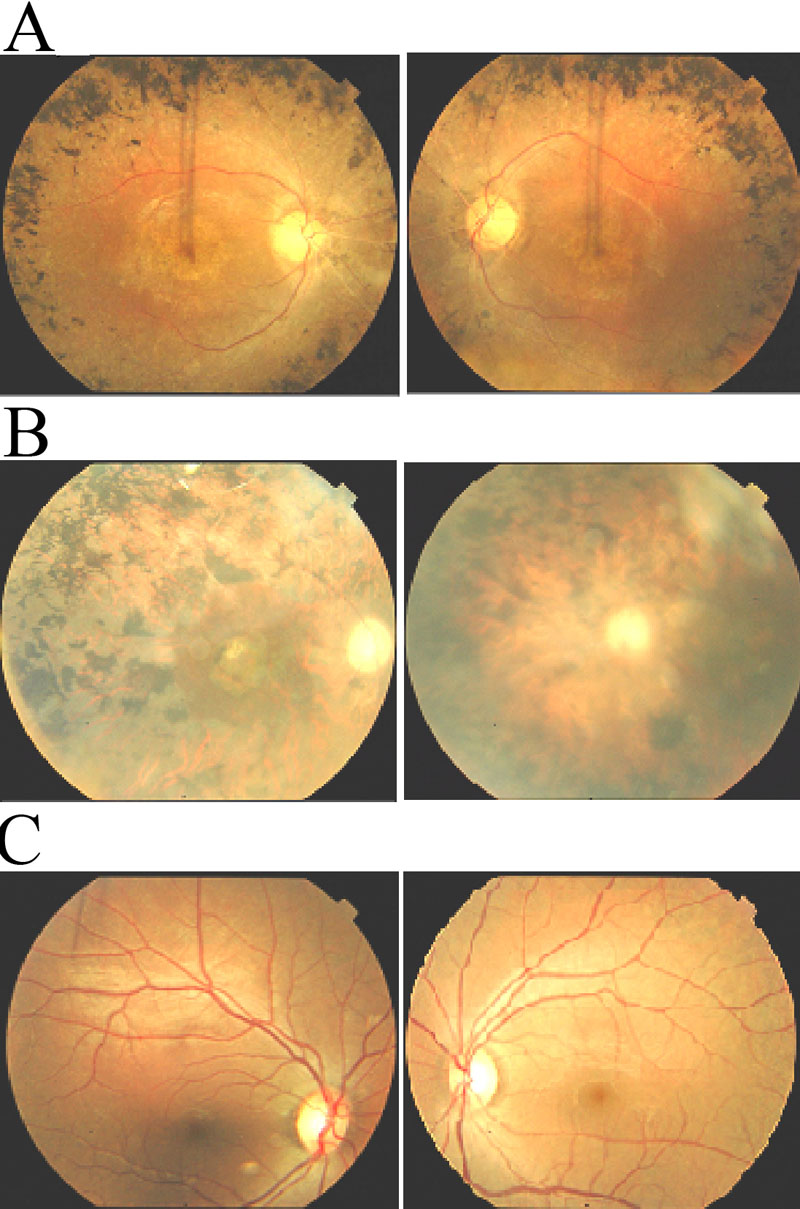
Fundus photographs of affected individuals of families PKFP161 and PKRP183. **A**: OD and OS of affected individual 17 of PKRP161 (age: 28 years). **B**: OD and OS of affected individual 7 of PKRP183 (age: 55 years). **C**: OD and OS of unaffected individual 19 of PKRP183 (age: 51 years). Photographs show peripheral fundus demonstrating several features associated with RP including a waxy pallor of the optic disc, attenuated arterioles and peripheral bone spicules. Visual acuity were recorded as 6/24, 6/24 (OD, OS) and 6/18, 6/18 (OD, OS) for individual 17 of PKRP161 and individual 7 of PKRP183, respectively. OD: Oculus Dexter (right eye); and OS: Oculus Sinister (left eye).

**Figure 3:**
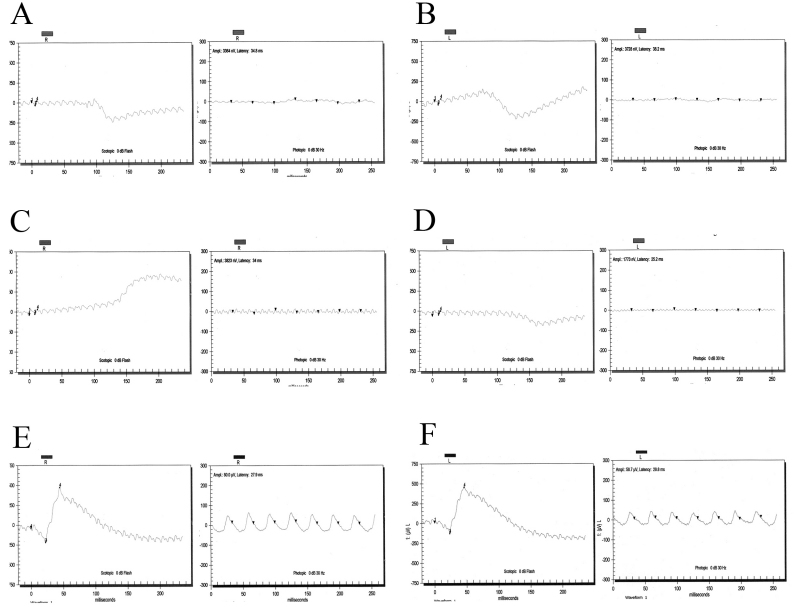
Electroretinography recordings of affected individuals of families PKFP161 and PKRP183. **A**: OD combined rod and cone response, and OD isolated cone response. **B**: OS combined rod and cone response; and OS isolated cone response of affected individual 17 of PKRP161. **C**: OD: combined rod and cone response, and isolated cone response. **D**: OS: combined rod and cone response, and isolated cone response of affected individual 7 PKRP183. **E**: OD: combined rod and cone response and isolated cone response. **F**: OS: combined rod and cone response and, isolated cone response of unaffected individual 18 of PKRP161. Affected individuals show loss of both rod and cone responses consistent with the diagnosis of Retinitis pigmentosa, whereas the unaffected has normal rod and cone responses. OD: Oculus Dexter (right eye); and OS: Oculus Sinister (left eye).

The families described in this study, PKRP161 and PKRP183, are from the Punjab province of Pakistan (Figure 1). All affected individuals underwent a thorough ophthalmologic examination that concluded that all affected individuals in the two families fulfill the diagnostic criteria of RP. Affected patients presented symptoms of night blindness in early childhood. Fundus examination of affected patients illustrated cardinal features of RP, namely arteriolar constriction, waxy pallor optic disc, and pigmentation on the peripheral as well as central retina (Figure 2). ERG recordings showed absence of both rod and cone responses; scotopic and photopic ERG responses were highly reduced or absent (Figure 3).

Exclusion analyses with closely spaced STR markers spanning the known/reported loci for arRP were completed. The results of these analyses strongly suggested that the pathogenic mutation in PKRP161 and PKRP183 resides in a region on chromosome 4p. For PKRP161 maximum two-point LOD scores of 3.77, 3.75, 3.63, and 3.66 were obtained with D4S3360, D4S2936, D4S3038, and D4S1614, respectively at θ=0 (Table 2). Similarly for PKRP183, two-point LOD scores of 3.08, 3.12, and 3.16 were obtained for markers D4S2936, D4S3038, and D4S1614, respectively at θ=0 (Table 2).

**Table 2:** Two-point LOD at different recombination fractions of families.

**Marker**	**cM**	**Mb**	**0**	**0.01**	**0.05**	**0.1**	**0.2**	**0.3**	**Z_max_**	**θ_max_**
**A:**										
D4S3360	0	0.11	3.77	3.69	3.39	3.01	2.17	1.43	3.77	0
D4S2936	1.48	0.69	3.75	3.66	3.35	3.04	2.12	1.38	3.75	0
D4S3038	1.48	1.09	3.63	3.56	3.25	2.86	2.04	1.21	3.63	0
D4S1614	4.74	2.64	3.66	3.58	3.27	2.89	2.07	1.27	3.66	0
D4S3023	8.24	4.3	- ∞	−1.57	−0.84	−0.31	−0.14	0.14	0.14	0.3
**B:**										
D4S3360	0	0.11	0.98	1.96	0.88	0.76	0.5	0.26	1.96	0.01
D4S2936	1.48	0.69	3.08	3.01	2.8	2.38	1.55	0.85	3.08	0
D4S3038	1.48	1.09	3.12	3.05	2.84	2.41	1.59	0.91	3.12	0
D4S1614	4.74	2.64	3.16	3.1	2.77	2.36	1.54	0.78	3.16	0
D4S3023	8.24	4.3	- ∞	−1.45	−0.75	−0.28	−0.12	0.1	0.1	0.3

**A**: PKRP161 and **B**: PKRP183 with alleles of chromosome 4p STR markers.

Haplotype analyses confirmed the linkage results, and visual inspection of these haplotypes localized the disease to chromosome 4p. We did not find any proximal recombination events in any affected individuals, whereas the distal boundary was defined by a recombination in affected individuals 17 and 22 of family PKRP161 and affected individual 12 of family PKRP183 at D4S3023. This places the disease interval in an 8.24 cM (4.3 Mb) region on chromosome 4pter-p16.2. Alleles for markers D4S3360, D4S2936, D4S3038, and D4S1614 are homozygous in affected individuals of the two families.

**Figure 4:**
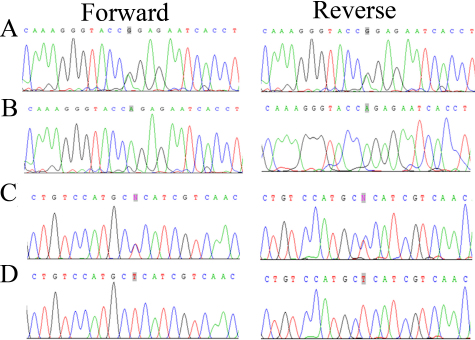
The sequence chromatograms of families PKRP161 and PKRP183. **A**: Unaffected individual 20 of PKRP161 heterozygous. **B**: Affected individual 17 homozygous for a single base change: c.1655G>A, leading to an Arginine to Glutamine substitution: p.R552Q. **C**: Unaffected individual 11 of family PKRP183 heterozygous. **D**: Affected individual 7 homozygous for a base change: c.1160 C>T, leading to Proline to Leucine substitution: p.P387L.

The linked region on chromosome 4pter-p16.2 harbors *PDE6β*, a gene that has previously been associated with the causality of arRP. We sequenced all coding exons, exon–intron boundaries, and the 5′ and 3′ untranslated regions of *PDE6β*. In PKRP161 we identified a missense mutation c.1655G>A (Figure 4), which results in an arginine residue substituted by a glutamine residue at position 552: p.R552Q. Similarly, we identified the novel missense variation c.1160 C>T in PKRP183 (Figure 4), which leads to a proline residue substituted by leucine at position 387: p.P387L. None of these two variations were present in 192 ethnically matched control chromosomes.

**Figure 5:**
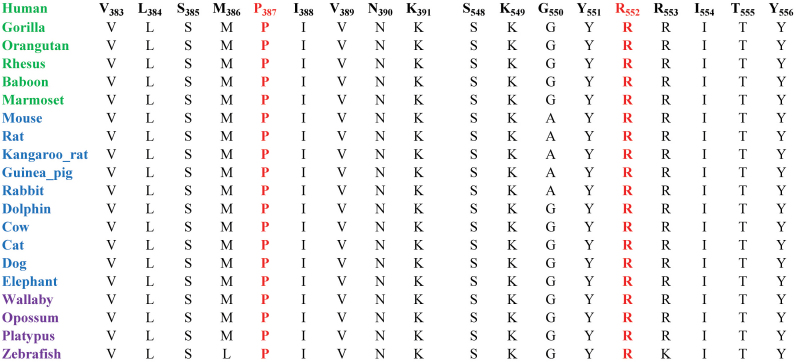
Amino Acid conservation of the P387 and R552 residues in other PDE6β orthologs. The arrows point to the amino acid residues P387 and R552, which are mutated in the affected individuals of families PKRP183 and PKRP161, respectively. Primates are colored green, placental mammals are blue, and vertebrates are purple.

As shown in Figure 5, both amino acid residues P387 and R552 are highly conserved among other PDE6β orthologs; therefore we next examined the possible impact of these two variations on PDE6β with SIFT and PolyPhen algorithms. SIFT predictions suggested that both p.P387L and p.R552Q substitutions will not be tolerated by the native three dimensional structure of PDE6β. The affected protein function score for p.P387L and p.R552Q were 0.01 and 0.02, respectively (scores <0.05 are predicted to be damaging). Likewise, position-specific score differences obtained from Polyphen suggested that both p.P387L and p.R552Q substitutions could potentially have deleterious effect on the PDE6β  structure, with a position- specific independent count scores of 3.09 and 2.24, respectively (scores >1 are predicted to be damaging).

## Discussion

Here, we report two consanguineous Pakistani families with multiple family members affected with arRP. Exclusion linkage analyses localized the critical interval to chromosome 4pter-p16.2 with significant LOD scores, and bidirectional sequencing identified two missense mutations, p.P387L and p.R552Q, that segregated with the disease phenotype in their respective families. These mutations were absent in ethnically matched control chromosomes and involved amino acids that are highly conserved in other PDE6β orthologs. These results strongly suggest that mutations in *PDE6β* are responsible for the disease phenotype in the consanguineous Pakistani families.

In this study we interrogated 125 families with closely spaced STR markers spanning the chromosome 4p locus harboring *PDE6β* but we only identified two families linked to the chromosome 4p locus, which suggests that mutations in *PDE6β* do not contribute significantly to the genetic load of arRP in the Punjab province of Pakistan.

Previously, Mou and colleagues [15,16] showed that the γ subunit of PDE6 interacts with at least two distinct sites on the catalytic subunit, and these interactions are regulated by cGMP binding to the regulatory cGMP-binding PDEs, Anabaena adenylyl cyclases, and *Escherichia coli* FhlA (GAF) domains. Of the two mutations identified in here, p.R552Q has previously been reported to be associated with arRP and resides in the catalytic domain of PDE6β, where it is presumed to perturb catalytic activity of the enzyme [17]. On the other hand p.P387L is believed to reside in the regulatory GAF domain, but the mechanism of the causality remains unknown.

Identification of pathogenic mutations reaffirms the allelic heterogeneity of *PDE6β* in the pathogenesis of RP, and future studies investigating the mechanism of the causality will help us better understand the molecular mechanism of the disease phenotype, which will lead to better treatments and novel therapeutics.
